# Lu^3+^/Yb^3+^ and Lu^3+^/Er^3+^ co-doped antimony selenide nanomaterials: synthesis, characterization, and electrical, thermoelectrical, and optical properties

**DOI:** 10.1186/1556-276X-8-141

**Published:** 2013-03-27

**Authors:** Younes Hanifehpour, Sang Woo Joo, Bong-Ki Min

**Affiliations:** 1 School of Mechanical Engineering, Yeungnam University, Gyongsan 712-749, South Korea; 2 Department of Applied Chemistry, Faculty of Chemistry, University of Tabriz, Tabriz, Iran; 3 Center for Research Facilities, Yeungnam University, Gyongsan 712-749, South Korea

**Keywords:** Co-doped, Nanomaterial, Luminescent, Electrical conductivity, Hydrothermal

## Abstract

Lu^3+^/Yb^3+^ and Lu^3+^/Er^3+^ co-doped Sb_2_Se_3_ nanomaterials were synthesized by co-reduction method in hydrothermal condition. Powder X-ray diffraction patterns indicate that the Ln_*x*_Ln^′^_*x*_Sb_2−2*x*_Se_3_ Ln: Lu^3+^/Yb^3+^ and Lu^3+^/Er^3+^ crystals (*x* = 0.00 − 0.04) are isostructural with Sb_2_Se_3_. The cell parameters were increased for compounds upon increasing the dopant content (*x*). Scanning electron microscopy and transmission electron microscopy images show that co-doping of Lu^3+^/Yb^3+^ ions in the lattice of Sb_2_Se_3_ produces nanorods, while that in Lu^3+^/Er^3+^ produces nanoparticles, respectively. The electrical conductivity of co-doped Sb_2_Se_3_ is higher than that of the pure Sb_2_Se_3_ and increases with temperature. By increasing the concentration of Ln^3+^ions, the absorption spectrum of Sb_2_Se_3_ shows red shifts and some intensity changes. In addition to the characteristic red emission peaks of Sb_2_Se_3_, emission spectra of co-doped materials show other emission bands originating from *f*-*f* transitions of the Yb^3+^ ions.

## Background

Nanosized semiconductor materials have drawn much research attention because their physical and chemical properties, due to size quantization effect, dramatically change and, in most case, are improved as compared with their bulk counterparts [1-3]. Rare earth-substituted compounds with various compositions have become an increasingly important research topic in diverse areas, such as luminescent device, light-emitting displays, biological labeling, and imaging [4-6], due to the introduction of dopant levels within the bandgap and modification of the band structure. In addition, significant efforts have been devoted to enhance the activity of wide bandgap photocatalysts by doping for environmental remediation [7,8]. Semiconductor selenides find applications as laser materials, optical filters, sensors, and solar cells. Antimony selenide, an important member of these *V*_*2*_*VI*_*3*_ compounds, is a layer-structured semiconductor of orthorhombic crystal structure and exhibits good photovoltaic properties and high thermoelectric power, which allows possible applications for optical and thermoelectronic cooling devices [9-11]. The research of impurity effects or doping agents on the physical properties of Sb_2_Se_3_ is interesting both for basic and applied research. Doping of some transition metal and lanthanide to the lattice of metal chalcogenides has been investigated [12-20]. The incorporation of large electropositive ions such as lanthanides into metal chalcogenide frameworks is expected to affect the electronic properties of that framework. In this work, we report the preparation, structural, electrical, and optical properties of Lu^3+^/Yb^3+^ and Lu^3+^/Er^3+^ co-doped antimony selenide via co-reduction method at hydrothermal condition.

## Methods

All chemicals were of analytical grade and were used without further purification. Gray selenium (1 mmol) and NaOH (5 mmol) were added to distilled water (60 mL) and stirred well for 10 min at room temperature. Afterwards, hydrazinium hydroxide (2 mL, 40 mmol), SbCl_3_ (1.98, 1.96, 1.94, and 1.92 mmol) and Ln_2_O_3_ (0.00, 0.01, 0.02, and 0.04 mmol) (Ln: Lu^3+^, Yb^3+^, Er^3+^) based on the molecular formula Ln_*x*_Ln^′^_*x*_Sb_2−2*x*_Se_3_ (0 ≤ *x* ≤ 0.04) were added, and the mixture was transferred to a 100-mL Teflon-lined autoclave. The autoclave was sealed, maintained at 180°C for 48 h, and then cooled to room temperature. The optimum conditions for this reaction are pH = 12, temperature = 180°C, and reaction time = 48 h. The black precipitate obtained was filtered and washed with ethanol and water. It was dried at room temperature. Yields for the products were 75% to 85%. Phase identification was performed by powder X-ray diffraction (XRD, D5000 Siemens AG, Munich, Germany) with Cu Kα radiation. Cell parameters were calculated using the Celref program (CCP14, London, UK) from powder XRD patterns, and reflections have been determined and fitted using a profile fitting procedure with the WinXPOW program (STOE & CIE GmbH, Darmstadt, Germany). The reflections observed in 2*θ* = 4° to 70° were used for the lattice parameter determination. The morphology of materials was examined by scanning electron microscopy (SEM, Hitachi S-4200, Hitachi High-Tech, Minato-ku, Tokyo, Japan). A linked ISIS-300 Oxford EDS detector (Oxford Instruments plc, Oxfordshire, UK) was used for elemental analyses. The high-resolution transmission electron microscopy (HRTEM) image and selected area electron diffraction (SAED) pattern were recorded by a Cs-corrected HRTEM (JEM-2200FS, JEOL Ltd., Akishima, Tokyo, Japan) operated at 200 kV. Photoluminescence measurements were carried out using a Spex FluoroMax3 spectrometer (HORIBA Jobin Yvon Inc., Edison, NJ, USA) after dispersing a trace amount of sample via ultrasound in distilled water. Four-point probe method was used for the measurement of electrical and thermoelectrical resistivity of samples. A small oven was needed for the variation of temperature of the samples from the room temperature to about 200°C (maximum). A small chip with 1-mm thickness and 7-mm length was used for this analysis.

## Results and discussion

**Figure 1:**
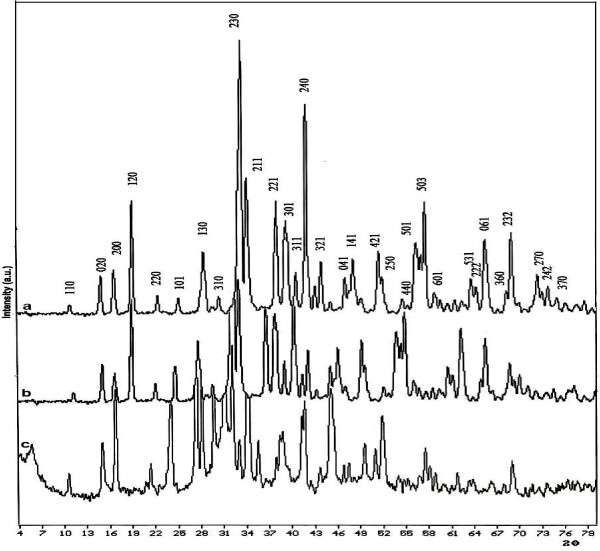
**Powder XRD pattern of Lu**_***x***_**Yb**_***x***_**Sb**_**2**−***x***_**Se**_**3**_**.** Curve **a**: *x* = 0.0, curve **b**: *x* = 0.04, and curve **c** = impurity phase.

The powder XRD patterns (Figure 1) of Lu_*x*_Yb_*x*_Sb_2−2*x*_Se_3_ samples indicate that the Lu^3+^/Yb^3+^ co-doped antimony selenide has the same orthorhombic structure as Sb_2_Se_3_ and that single-phase Sb_2_Se_3_ is retained at lower doping concentrations of Lu^3+^/Yb^3+^. All the peaks in Figure 1 can be attributed to the orthorhombic phase of Sb_2_Se_3_ with Pbnm space group and lattice parameters *a* = 11.62 Å, *b* = 11.76 Å, and *c* = 3.95 Å (JCPDS card file 72–1184). For doping levels higher than *x* = 0.04 for Lu^3+^ and Yb^3+^, additional unknown phases were observed (curve c of Figure 1). In the case of Lu^3+^/Er^3+^ co-doped compounds, the intensity of some peaks has been changed, and for doping levels higher than of *x* = 0.04 for Lu^3+^ and Er^3+^, additional unknown phases were also observed (see Additional file 1).

**Figure 2:**
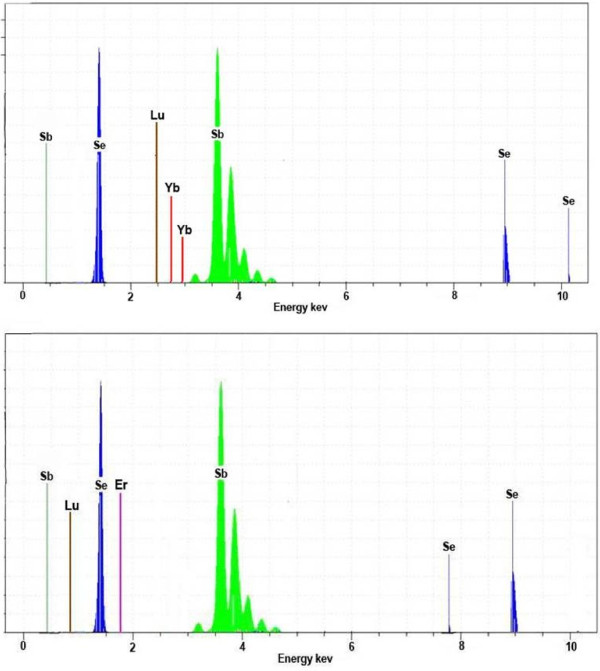
**EDX patterns of Ln**_***x***_**Ln**′_***x***_**Sb**_**2**−**2*x***_**Se**_**3 **_**compounds.**

In addition, a little shift toward the low angle was seen in the diffraction peaks of the co-doped Sb_2_Se_3_ compared with those of the undoped Sb_2_Se_3_ nanocrystals. This suggests that the larger lanthanide ions substitute the antimony ions, resulting in increased lattice constants. As expected, the EDX and ICP analyses of the product confirm the ratio of Sb/Se/Ln/Ln^′^ (see Figure 2).

**Figure 3:**
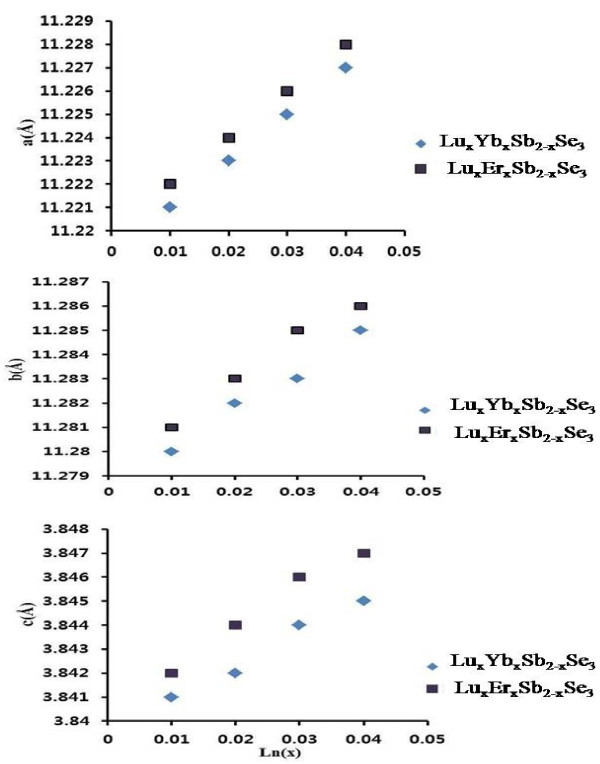
**The lattice constants of co**-**doped Sb**_**2**_**Se**_**3 **_**dependent upon Ln**^**3 **+^**doping on Sb**^**3 **+^**sites.**

The cell parameters of the synthesized materials were calculated from the XRD patterns. With increasing dopant content (*x*), the lattice parameters were increased for these materials, as shown in Figure 3. This trend is similar to the previous reported Ln-doped Sb_2_Se_3_ compounds [16-20].

**Figure 4:**
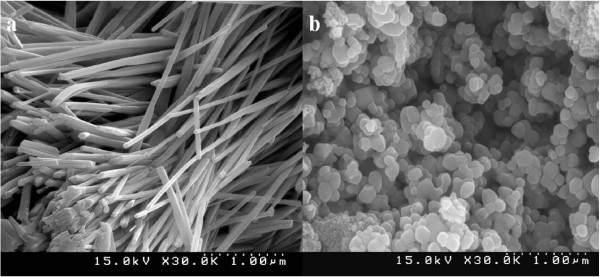
**SEM images of co-doped antimony selenide.** (**a**) Lu_0.04_Yb_0.04_Sb_1.92_Se_3_ nanorods (**b**) Lu_0.04_Er_0.04_Sb_1.92_Se_3_ nanoparticles.

Figure 4a shows SEM images of Lu_0.04_Yb_0.04_Sb_1.92_Se_3_ nanorods with 3-μm lengths and thicknesses of 70 to 200 nm. Co-doping of Lu^3+^ and Yb^3+^ into the structure of Sb_2_Se_3_ does not change the morphology of the Sb_2_Se_3_ nanorods, but doping of Lu^3+^ and Er^3+^ into the structure of Sb_2_Se_3_ changes the morphology from rods to particles. The diameter of Lu_0.04_Er_0.04_Sb_1.92_Se_3_ particles is around 25 nm (Figure 4b).

**Figure 5:**
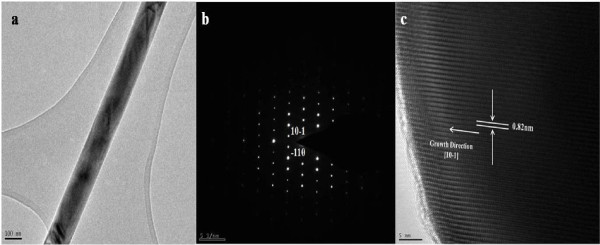
**TEM (a), SAED pattern (b), and HRTEM image (c) of Lu**_**0**.**04**_**Yb**_**0**.**04**_**Sb**_**1**.**92**_**Se**_**3 **_**nanorods.**

Figure 5a shows TEM image of as-prepared Lu_0.04_Yb_0.04_Sb_1.92_Se_3_ nanorods. The SAED pattern and typical HRTEM image recorded from the same nanorods of Lu_0.04_Yb_0.04_Sb_1.92_Se_3_ is shown in Figure 5b,c. The crystal lattice fringes are clearly observed, and the average distance between the neighboring fringes is 0.82 nm, corresponding to the [1-10] plane lattice distance of the orthorhombic-structured Sb_2_Se_3_, which suggests that Lu_0.04_Yb_0.04_Sb_1.92_Se_3_ nanorods grow along the [1] direction. The HRTEM image and SAED pattern are the same for Sb_2_Se_3_ and show similar growth direction (see the Additional file 1).

**Figure 6:**
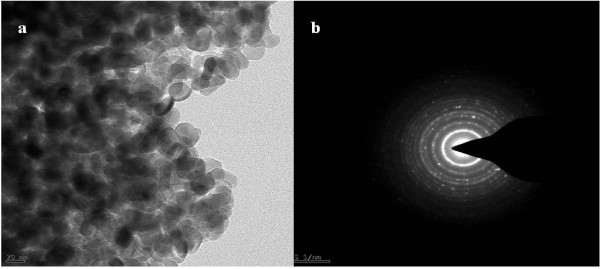
**TEM (a) and SAED pattern (b) of Lu**_**0**.**04**_**Er**_**0**.**04**_**Sb**_**1**.**92**_**Se**_**3 **_**nanoparticle.**

Figure 6a,b shows the TEM image and SAED patterns of Lu_0.04_Er_0.04_Sb_1.92_Se_3_ nanoparticles obtained in ethanol/water media that confirms the result through SEM images and shows high crystallinity of the sample.

**Figure 7:**
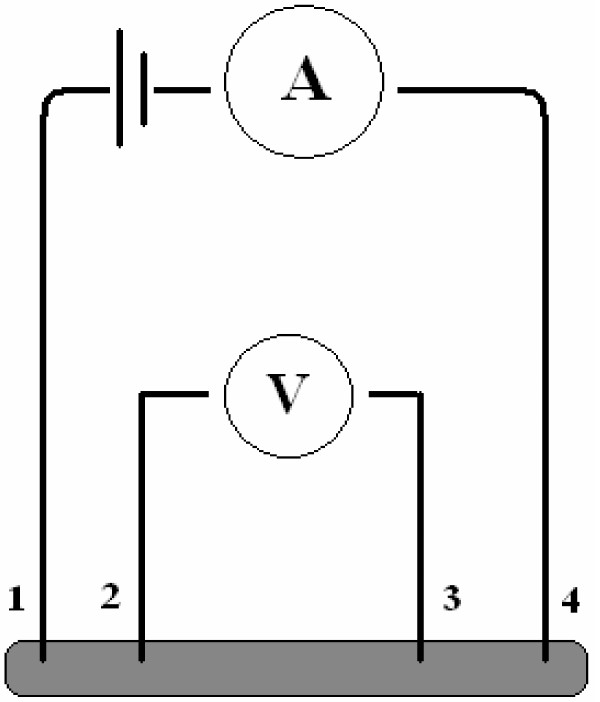
**Schematic of four-point probe.**

In doped semiconductors, two types of emissions are responsible for dopant (impurity) luminescence. One can be observed only upon direct excitation of the dopant. The other type is obtained if energy transfer from host to dopant occurs. Binary compounds such as Sb_2_Se_3_ and its alloys are thermoelectric materials with layered crystalline structures. These materials have been investigated for the direct conversion of thermal energy to electric energy, and they are specially used for electronic refrigeration [9]. The four-point probe method was used for the measurement of electrical and thermoelectrical resistivity of samples (Figure 7).

**Figure 8:**
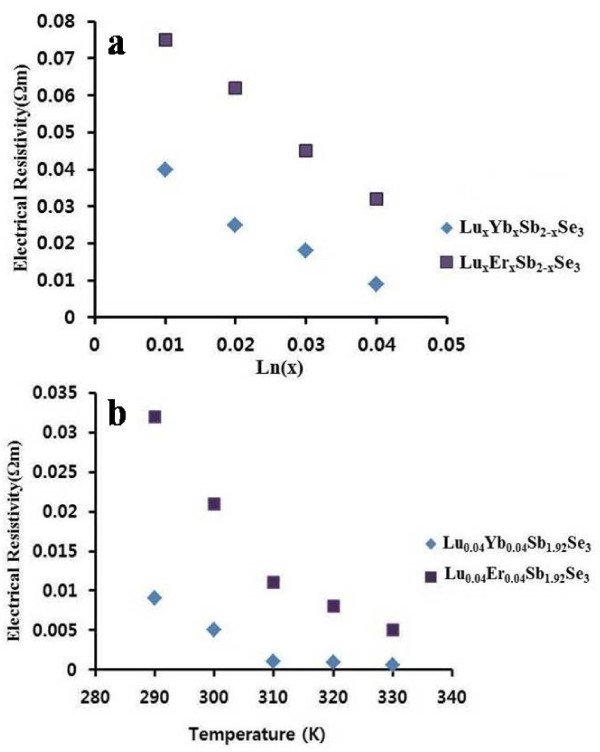
**Electrical (a) and thermoelectrical (b) resistivity of co-doped Sb**_**2**_**Se**_**3 **_**compounds.**

At room temperature, the electrical resistivity of pure Sb_2_Se_3_ was of the order of 0.2 Ω·m; in the case of Lu_0.04_Yb_0.04_Sb_1.92_Se_3_, the minimum value of electrical resistivity is 0.009 Ω·m, and for Lu_0.04_Er_0.04_Sb_1.92_Se_3_, it is 0.032 Ω·m. With the increase in lanthanide concentration, the electrical resistivity of synthesized nanomaterials decreased obviously (Figure 8a).

The temperature dependence of the electrical resistivity for co-doped Sb_2_Se_3_ nanomaterials between 290 and 350 K is shown in Figure 8b. Electrical resistivity decreases linearly with temperature, and the minimum value for Lu_0.04_Yb_0.04_Sb_1.92_Se_3_ was measured as 0.0006 Ω·m and for Lu_0.04_Er_0.04_Sb_1.92_Se_3_ as 0.005 Ω·m. Two factors that include the overlapping of wave functions of electrons in doped Sb_2_Se_3_ and that acting as a charge carrier due to lanthanide atomic structure (having empty f orbitals) are important reasons for decreasing electrical resistivity. The obtained data shows higher electrical resistivity for co-doped samples in comparison with doped samples in the case of Lu^3+^, Yb^3+^ and Er^3+^ doped Sb_2_Se_3_[16,17]. The measurements indicate that the co-doping materials have higher electrical and thermoelectrical conductivity than the doped compounds in spite of lower lanthanide content [16-20]. Comparing both doped and co-doped data, the combining energy levels of the two lanthanides and the overlapping of wave functions of electrons in two different lanthanides are responsible for the difference between the obtained results. Among the co-doped compounds, Lu^3+^/Yb^3+^-doped Sb_2_Se_3_ has the higher electrical conductivity.

**Figure 9:**
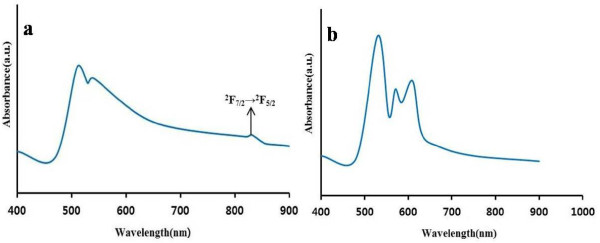
**Absorption spectra of co-doped antimony selenide at room temperature.** (**a**) Lu_0.04_Yb_0.04_Sb_1.92_Se_3_ (**b**) Lu_0.04_Er_0.04_Sb_1.92_Se_3_.

UV–vis spectra of Lu_0.04_Yb_0.04_Sb_1.92_Se_3_ are shown in Figure 9a. The absorption spectra reveal the existence of Sb_2_Se_3_ and Lu^3+^ ions (in the visible domain) and Yb^3+^ ions in the near-IR domain. By increasing the concentration of Ln^3+^ ions, the absorption spectrum of Sb_2_Se_3_ shows red shifts and some intensity changes (see Additional file 1). The Lu^3+^ ion has no excited 4*f* levels; therefore, the peaks between 500 and 600 nm can be assigned to the ionization of Lu 5*d* orbitals and lattice of Sb_2_Se_3_.[21,22], and the peak at 830 nm can be assigned to the ^2^*F*_7/2_→^2^*F*_5/2_ transition (*f*-*f* transitions) of the Yb^3+^ ions [23].

For Lu_0.04_Er_0.04_Sb_1.92_Se_3_, the transition of the Er^3+^ ions is not observed because of instrument limitation. The peaks between 500 and 620 nm can then be assigned to the lattice of Sb_2_Se_3_ (Figure 9b). The difference between absorption patterns of compounds is related to various defects created in the lattice. There is a red shift in the doped materials in comparison with pure Sb_2_Se_3_ because of the smaller nanoparticles of Sb_2_Se_3_, in which the bandgap is higher than the doped nanomaterials [24,25]. It is well known that the fundamental absorption can be used to determine the nature and value of the optical bandgap of the nanoparticles. The bandgap energies of samples were estimated from the absorption limit. The calculated bandgap is 2.43 eV for Lu_0.04_Yb_0.04_Sb_1.92_Se_3_ and 2.36 eV for Lu_0.04_Er_0.04_Sb_1.92_Se_3_.

**Figure 10:**
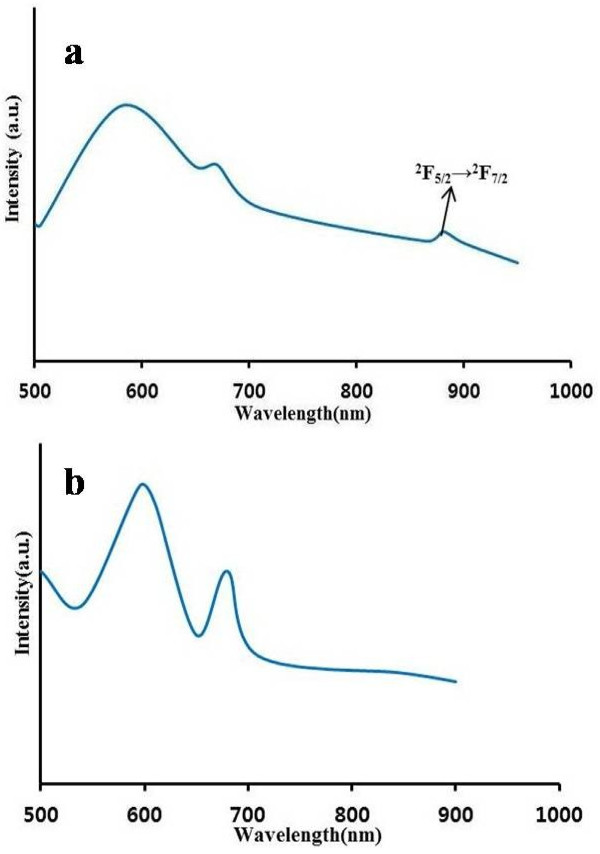
**Emission spectra for co-doped antimony selenide at room temperature (*λ***_**exc **_**=470 nm).** (**a**) Lu_0.04_Yb_0.04_Sb_1.92_Se_3_ (**b**) Lu_0.04_Er_0.04_Sb_1.92_Se_3_.

Figure 10a exhibited the room-temperature photoluminescence emission spectra of Lu_0.04_Yb_0.04_Sb_1.92_Se_3_. The Lu^3+^ 5*d*-4*f* luminescence is almost completely quenched at temperatures *T* > 200 K. The Lu^3+^ ion has no excited 4*f* levels, and therefore, thermal quenching of Lu^3+^ 5*d*-4*f* luminescence cannot have been caused by nonradiative transitions to 4*f* levels and should be attributed to the thermally activated ionization of 5*d* electrons to the conduction band [21,22]. The peaks at 500 to 700 nm can then be assigned to the crystal structure of Sb_2_Se_3_, and its defects and the band at 880 nm is related to ^2^*F*_5/2_→^2^*F*_7/2_ transition of Yb^3+^ions.

In case the of Lu_0.04_Er_0.04_Sb_1.92_Se_3_, intra-4*f* Er^3+^ transitions of the ^4^I_11/2_ and ^4^I_13/2_ levels to the ground state (^4^I_15/2_) are expected around 1.54 μm. These could, however, not be determined due to equipment limitations [24]. Therefore, emission bands at 550 to 700 nm are related to the crystal structure of Sb_2_Se_3_ (Figure 10b). The optical properties of co-doped compounds considering absorbance and photoluminescence spectra show similar *f*-*f* transitions in the case of Yb-doped materials and similar results for Lu- and Er-doped materials as obtained for Ln-doped Sb_2_Se_3_. We expect that these materials can be good candidates as novel photocatalysts due to their modified bandgaps by doping with lanthanides. Indeed, doping is the best way for the modification of semiconductors for special uses such as photocatalysts in order for the degradation of azo dye and organic pollutant to take place.

## Conclusions

New thermoelectric Ln_2*x*_Sb_2−2*x*_Se_3_ (Ln: Lu^3+^/Yb^3+^ and Lu^3+^/Er^3+^)-based nanomaterials were synthesized by a simple hydrothermal method. The cell parameters were increased for compounds upon increasing the dopant content (*x*). According to the SEM and TEM images, different morphologies were seen in co-doped Sb_2_Se_3_. The HRTEM image and SAED pattern show similar growth [1] directions for Lu^3+^/Yb^3+^ co-doped like Sb_2_Se_3_ nanorods. Lanthanide doping promotes the electrical conductivity of Sb_2_Se_3_ as well as thermoelectrical conductivity. UV–vis absorption and emission spectroscopy reveals mainly the electronic transitions of the Ln^3+^ ions in the case of Yb^3+^-doped nanomaterials.

## Competing interests

The authors declare that they have no competing interests.

## Authors’ contributions

YH carried out the experiments and drafted the manuscript. SWJ directed the study and provided the analyses. BM carried out the experimental analysis. All authors read and approved the final manuscript.

## Supplementary Material

Additional file 1 **XRD patterns of Lu**_***x***_**Er**_***x***_**Sb**_**2−2*x***_**Se**_**3**_**, TEM, HRTEM images, SAED pattern of Sb**_**2**_**Se**_**3**_** nanorods, absorption spectra of Lu**_**0.02**_**Yb**_**0.02**_**Sb**_**1.96**_**Se**_**3**_**, Lu**_**0.01**_**Yb**_**0.01**_**Sb**_**1.98**_**Se**_**3**_**, and Lu**_**0.02**_**Er**_**0.02**_**Sb**_**1.96**_**Se**_**3**_** are provided. Figure S1.** Powder X-ray diffraction pattern of Lu_*x*_Er_*x*_Sb_2−*x*_Se_3_ (*x* = 0.02). **Figure S2.** Powder X-ray diffraction pattern of Lu_*x*_Er_*x*_Sb_2−*x*_Se_3_ (*x* = 0.04). **Figure S3.** Powder X-ray diffraction pattern of unknown Lu_*x*_Er_*x*_Sb_2−*x*_Se_3_ phase. **Figure S4.** TEM image of Sb_2_Se_3_ nanorods. **Figure S5.** HRTEM image of the Sb_2_Se_3_ nanorods. **Figure S6.** SAED Pattern of the Sb_2_Se_3_ nanorods. The SAED zone axis is [1]. **Figure S7.** Absorption spectra of Lu_0.02_Yb_0.02_Sb_1.96_Se_3_ nanorods at room temperature. **Figure S8.** Absorption spectra of Lu_0.01_Yb_0.01_Sb_1.98_Se_3_ nanorods at room temperature. **Figure S9.** Absorption spectra of Lu_0.02_Er_0.02_Sb_1.96_Se_3_ nanoparticles at room temperature. (DOC 3322 kb) Click here for file
