# Supplementary material for: Lu3+/Yb3+ and Lu3+/Er3+ co-doped antimony selenide nanomaterials: synthesis, characterization, and electrical, thermoelectrical, and optical properties
Source: Nanoscale Res Lett. 2013 Mar 27;8(1):141. doi: 10.1186/1556-276X-8-141 (PMC3618067; doi:10.1186/1556-276X-8-141)
Supplement: Additional file 1 — XRD patterns of LuxErxSb2−2xSe3, TEM, HRTEM images, SAED pattern of Sb2Se3 nanorods, absorption spectra of Lu0.02Yb0.02Sb1.96Se3, Lu0.01Yb0.01Sb1.98Se3, and Lu0.02Er0.02Sb1.96Se3 are provided. Figure S1. Powder X-ray diffraction pattern of LuxErxSb2−xSe3 (x = 0.02). Figure S2. Powder X-ray diffraction pattern of LuxErxSb2−xSe3 (x = 0.04). Figure S3. Powder X-ray diffraction pattern of unknown LuxErxSb2−xSe3 phase. Figure S4. TEM image of Sb2Se3 nanorods. Figure S5. HRTEM image of the Sb2Se3 nanorods. Figure S6. SAED Pattern of the Sb2Se3 nanorods. The SAED zone axis is [1]. Figure S7. Absorption spectra of Lu0.02Yb0.02Sb1.96Se3 nanorods at room temperature. Figure S8. Absorption spectra of Lu0.01Yb0.01Sb1.98Se3 nanorods at room temperature. Figure S9. Absorption spectra of Lu0.02Er0.02Sb1.96Se3 nanoparticles at room temperature. (DOC 3322 kb) [file 1556-276X-8-141-S1.doc]

**Supplementary data**

**
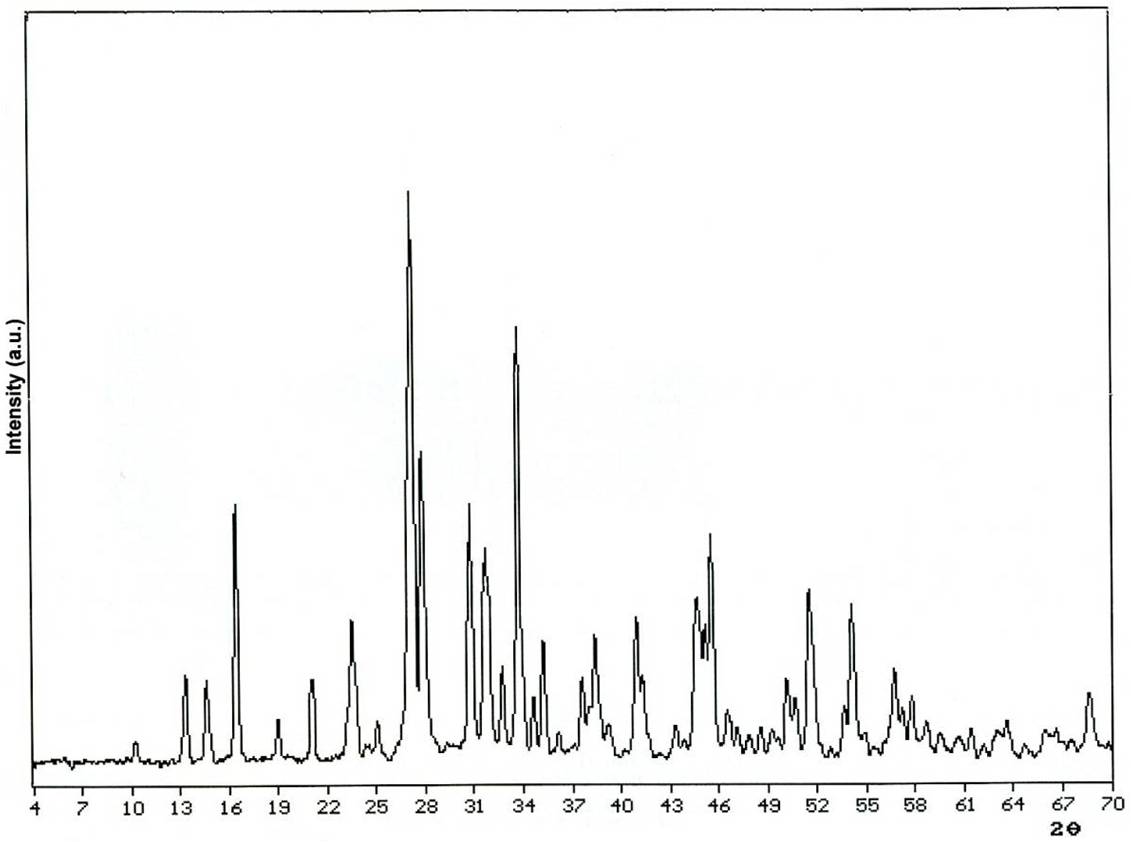
**

Fig.1. Powder X-ray diffraction pattern of Lu_x_Er_x_Sb_2–x_Se_3_ (x = 0.02)

**
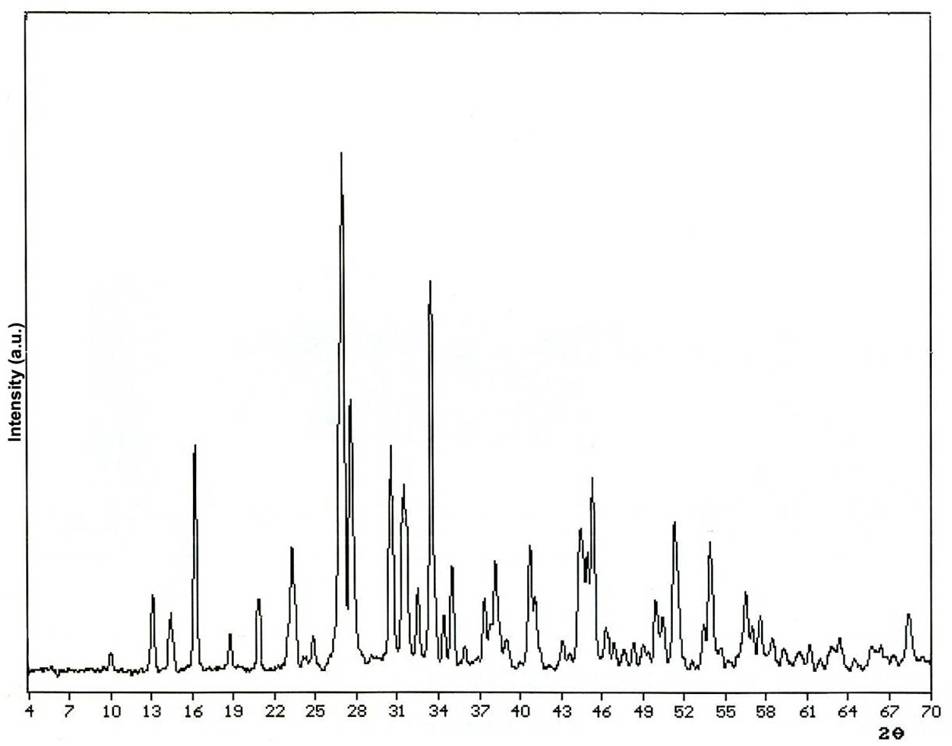
**

Fig.2. Powder X-ray diffraction pattern of Lu_x_Er_x_Sb_2–x_Se_3_ (x = 0.04)

**
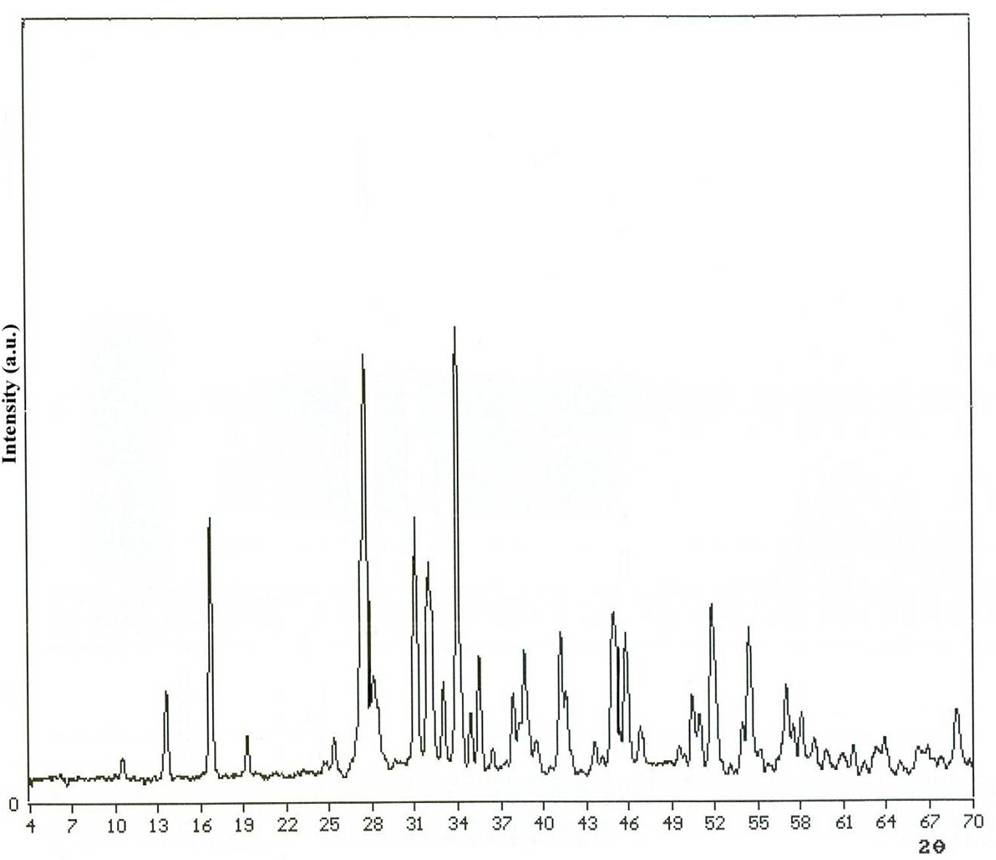
**

Fig.3. Powder X-ray diffraction pattern of unknown Lu_x_Er_x_Sb_2–x_Se_3_ phase.


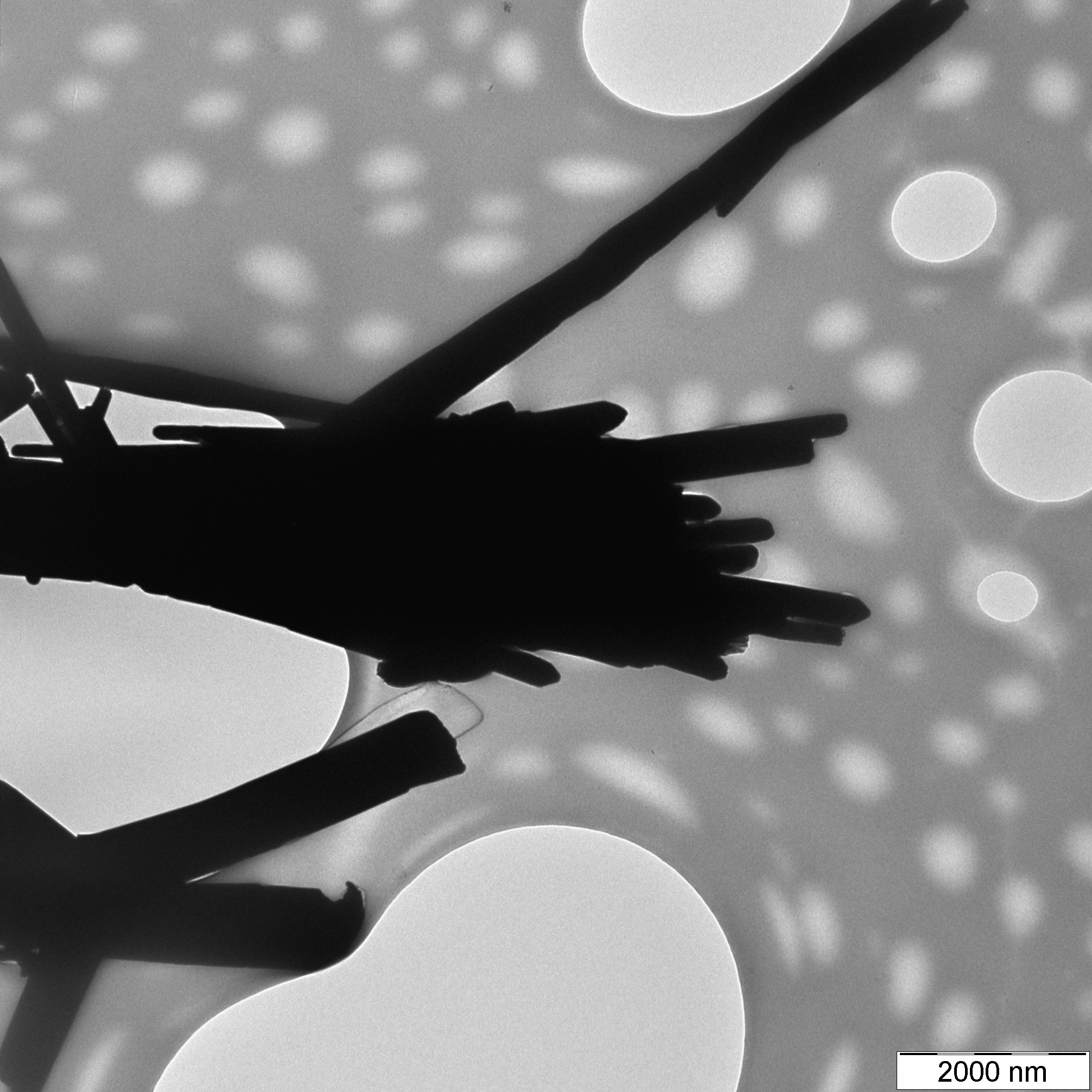


Fig.4.TEM image of Sb_2_Se_3_ nanorods.


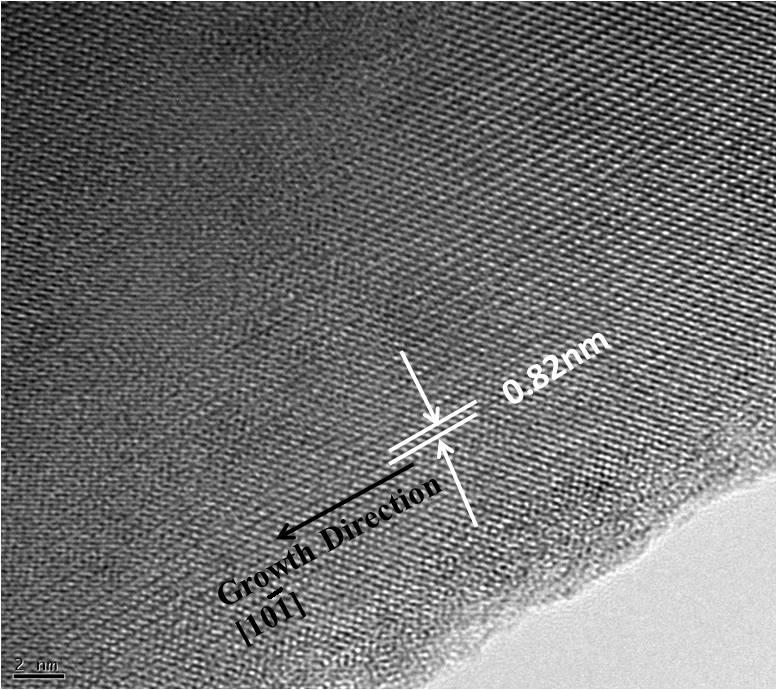


Fig.5.HRTEM image of the Sb_2_Se_3_ nanorods.


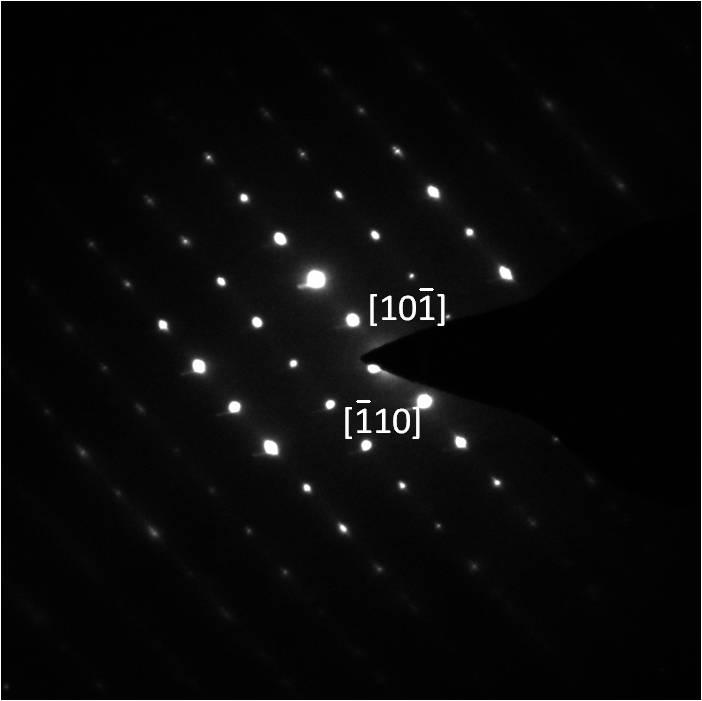


Fig.6. SAED Pattern of the Sb_2_Se_3_ nanorods. The SAED zone axis is [10-1].


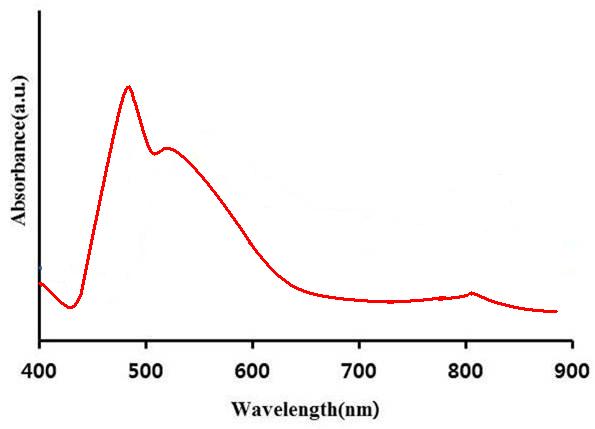


Fig 7.Absorption spectra of Lu_0.02_Yb_0.02_Sb_1.96_Se_3_ nanorods at room temperature


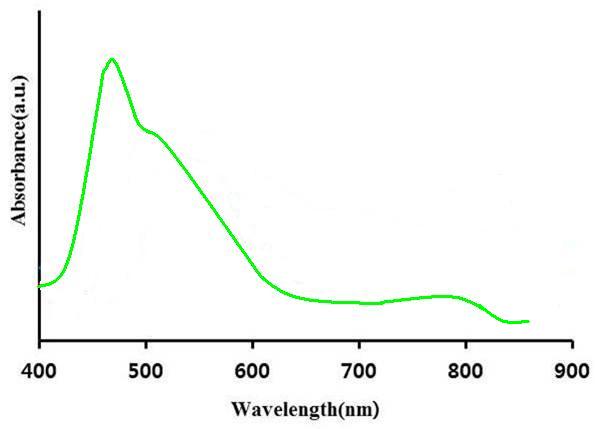


Fig 8.Absorption spectra of Lu_0.01_Yb_0.01_Sb_1.98_Se_3_ nanorods at room temperature


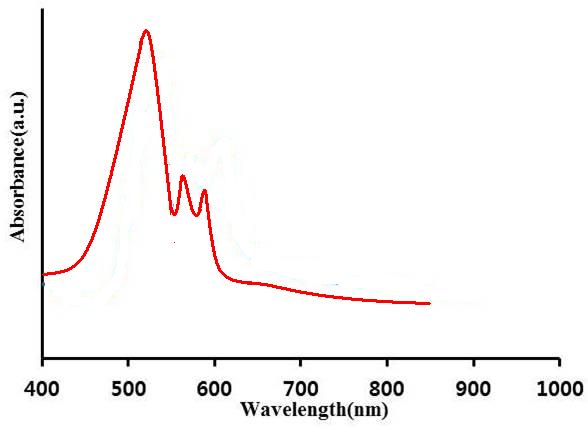


Fig9.Absorption spectra of Lu_0.02_Er_0.02_Sb_1.96_Se_3_ nanoparticles at room temperature
